# An Exploratory Analysis of the Role of Religion in Colorectal Cancer Screening among Safety-Net Clinic Patients

**DOI:** 10.23937/2469-5858/1510058

**Published:** 2019

**Authors:** Crystal Y Lumpkins, K Allen Greiner, Christine Daley, Jannette Berkley-Patton, Jinxiang Hu, Shana Palla

**Affiliations:** 1 Department of Family Medicine, University of Kansas Medical Center, Kansas City, USA; 2 Department of Bioinformatics, University of Missouri-Kansas City, School of Medicine, Kansas City, USA; 3 Department of Biostatistics, University of Kansas Medical Center, Kansas City, USA

**Keywords:** Religion, Religious involvement, Colon cancer screening, Low-Income patients, Minorities

## Abstract

Colorectal cancer (CRC) incidence among low income populations is disproportionate when compared to the general population. Cancer screening studies show religion as a potentially influential factor in individual screening. The present study was an exploratory analysis of religious involvement (RI) among older safety-net clinic patients who participated in 90-day follow up calls during an intervention trial. Results show RI among participants (n = 185) did not significantly predict nor was associated with screening for CRC (OR = 1.36, p = 0.35). The percentage of participants that self-identified as being highly religious differed across racial/ethnic groups (25% of Non-Hispanic Whites, 22% of Hispanics were highly religious when compared to 52% of Non-Hispanic Blacks). These findings raise questions about the use of religious appeals as part of health promotion for CRC screening and religious involvement among low-income patient populations. Varied religious beliefs across groups suggest while there may be room for including religion in CRC screening promotion targeted to some patients from low income groups, this appeal would not be suitable for other low-income patient sub-populations.

## Introduction

### Colorectal cancer among low-income and minority populations

Colorectal cancer (CRC) is the second leading cause of cancer death in the U.S. and third most common cancer in men and women [1]. Minority and low-income populations fare worse than the general population and experience a greater incidence and mortality from colorectal (CRC) cancer [2,3]. Colorectal cancer is one of the most curable cancers if detected early. Among the most common screenings that health organizations recommend for CRC include the yearly fecal occult blood test (FOBT); Sigmoidoscopy given every 3–5 years and colonoscopy every 10 years for individuals > 50 who are not at high risk [4–6]. Although these screening tests are available; are covered by Medicaid and Medicare and most insurances; and have shown to impact adherence, CRC incidence and death rates among low income and minority populations in the U.S. remain disproportionate [7–9]. Disparities exist in part because of differential access to medical care and screening tests due to a lack of insurance [10,11], low adherence to screening test recommendations [12], mistrust of medical personnel [13,14], limited knowledge and health literacy [15–17] and cultural and behavioral health preferences and beliefs that facilitate CRC development and disease [18,19].

Overcoming such barriers may remove obstacles for seeking preventive care, enhance follow up and decrease CRC morbidity and mortality. Emerging research shows religion as an influence on health behavior and health outcome among low-income populations [20]. Through the multi-dimensional aspects of religion, health promotion may be tailored and promulgated to individuals who have adopted religion as a central part of life. Religion, in the aspect of involvement becomes a health promotion tool to impact cancer screening uptake among low-income safety-net populations.

### Religious involvement and CRC prevention among low-income and minority populations

Religion is an integral part of life for many Americans and often serves as a guide for spiritual and emotional well-being and avenue for social involvement. Religion also has signified an important factor in the lives of low-income populations in the U.S. [20]. When SES (socioeconomic status) was compared between lower-income groups and higher-income groups, individuals in the lower income groups showed “stronger religious belief than their higher-income counterparts” [20]. Collectively, these groups also were likely to adopt more traditional worship practices at theologically conservative institutions compared to higher-income groups where more liberal institutions were the preference for worship activities. Religion as an avenue to express strong religious beliefs through worship services and other religious activities underscores the relevance in health screening promotion targeting this population.

Religious involvement has shown as a positive association with health outcome and also a factor to consider when addressing cancer screening uptake and adherence [21]. Religious involvement, a multidimensional religious concept, includes both behavioral and functional aspects and provides a framework to explain or predict whether an individual’s connection with a social network (e.g. church, synagogue) will result in positive or negative outcomes. Studies indicate religious involvement protects against negative health outcomes [22,23], associated with positive outcomes [24] and religious affiliation and attendance have also shown as significant predictors of health outcome variables [25].

While research on the role of religion among low-income populations is emerging, there are still several unknowns about the associations and effects of religion on screening behavior outcome. Given that religious involvement is positively associated with cancer screening behavior among many low-income and vulnerable populations [26] and health outcome [27], it is plausible there may be a significant relationship between religious involvement and CRC screening behavior outcome among low income patient populations. This study aimed to address a gap in religious and public health literature between CRC screening behavior and religious involvement and the relationship between these items in a low-income patient population. Specifically, we examined associations between an individual’s religious involvement and screening for colon cancer (via FOBT or Colonoscopy) at 1) A 90-day follow up period following a CRC screening intervention; 2) Whether there was any predictive value of religious factors in CRC screening outcome at 90 days follow up among this population. To our knowledge, this is the first study that explored the association of religious involvement and CRC screening among a safety-net clinic patient population.

## Materials and Methods

### Settings and sample participants

Exploratory data for this study were obtained from a subset of low-income men and women (n = 185) who participated in the Touch to Screen Project (Touch2Screen), a randomized controlled trail aimed at increasing colorectal cancer screening rates among low-income safety net clinic patients in Wyandotte, County, Kansas from 2009–2011 [28]. The study assessed the efficacy of a novel tailored touch screen computer intervention delivered in primary care settings on low-cost touch screen computers through multimedia audio-narrative and video messages. Testing included generic information versus a multimedia tailored intervention that addresses each participant’s screening test modality preference.

The current study was not randomized but included participants from both arms of the parent study. The goal of this secondary study was to query them about their religious beliefs and religious activities at 90-days follow up after the intervention.

The population of the county sampled comprises of primarily five racial groups: Non-Hispanic Whites (42.7%), Non-Hispanic Blacks (25.1%), Hispanics (27.1%), Asian (3.4%) and American Indian (1.4%) (US Census Bureau).

Eligible participants for the parent study and the present study had to be uninsured or on Medicaid, meet low-income eligibility criteria (< 150% of household federal poverty level), be 50 years of age or older and not up-to-date with screening, and have an appointment for an office visit on the day of recruitment. Individuals with an acute medical illness, current gastrointestinal bleeding, a history of adenomatous polyps, CRC, an inherited polyposis/non-polyposis syndrome, inflammatory bowel disease, or a first degree relative with CRC prior to age 60 were excluded. Individuals with another household member enrolled in the study were also ineligible, as were individuals who had previously enrolled in the study. Recruitment at the six clinics occurred on a rotating basis, randomized into two separate 6-week blocks over a 24-month period to increase the opportunity to enroll those seen infrequently and to minimize the effect of any seasonal bias on recruitment or results. During recruitment times, research staff screened all available patients for eligibility. We recruited only patients receiving care in these primary care settings, both to assure that our sample was representative of low-income individuals typically seen in urban core primary care clinics.

At 90-day follow up, a subset of enrolled patients (n = 185) were given the religious involvement survey conducted as part of the Touch2Screen study. This subset sample served as an exploratory dataset and thus provided research study staff a representative sampling of the larger data set toward the end of follow-up data collection (March 2010 to May 2010). Given the size the larger sample (n = 417) and the time frame that data were collected from all patients (September 2008 to May 2010), the last cycle of follow up data (90-day follow up) is representative for the present study. Baseline data items included participant demographics, race, thoughts about CRC, information about CRC and screening, barriers to screening and screening preference (FOBT or Colonoscopy). Surveys were conducted over the phone with study staff and were completed within 15 to 20 minutes. Participants were also given a gift card for completion of the survey.

### Study measures

Religious involvement behavioral items (religious affiliation, church attendance) and functional items (connection to a higher power; beliefs about a higher power’s involvement with physical health) were included in the 90-day follow up survey. Religious involvement measures were assessed using three items [29]; religion questions related to a higher power and the impact on physical health were taken from formative data collected through meetings with community members and qualitative research with minority and ethnic populations [30] and existing data [31].

The first religious involvement measure, religious affiliation, was categorized as Protestant, Catholic, Buddhist, Muslim, Jewish, Atheist, Agnostic or none. The second measurement for religious involvement, church attendance, was self-reported frequency of church attendance and was categorized as high (attending service every week); moderate (attending a few times a month or once a month); low (occasionally/a few times a year) or never. In addition, participants were asked one item about how often they participated in activities at their place of worship. The third religious measure asked whether the participant considered himself or herself a spiritual person; the remaining questions were used to assess beliefs about the linkage between religion and physical health outcome and included: how religion affected decision making about health; whether there was a reliance on a higher power to stay healthy through prayer; if there was a reliance on God or a higher power through prayer *and* medicine to stay healthy.

Participants who reported 1) Religious affiliation or 2) High or moderate attendance and 3) Reported as a spiritual person 4) and strongly agreed, agreed and somewhat agreed that they relied on a higher power or religion to make health decisions was categorized as high religious involvement. Since there were 23 participants who were asked, but did not respond to the religious involvement survey questions, we used two approaches for creation of the high religious involvement classification. The first approach conservatively imputed a non-high religious involvement classification for the non-responders. The second approach excluded non-responders from the analysis altogether.

The primary outcome variable was whether the participant had screened for CRC with either FOBT (fecal occult blood test) or colonoscopy at the 90-days follow up. Participants were subsequently grouped as “no CRC screening at 90 days” and “CRC screening at/or before 90 days”.

### Data analysis

Logistic regression models were used to examine the relationship between variables at baseline and CRC screening completion at 90 days follow up. Because our interest was in the independent effect of religious involvement impact on colorectal cancer screening, a variable coding for the participant’s intervention status was included in the model to control for the treatment arm. A second set of models was constructed to examine the effect of (religious involvement) church attendance, religious beliefs and CRC screening controlling for potential confounders. The covariates examined were race (Hispanic, Non-Hispanic White, Non-Hispanic Black; Non-Hispanic other); marital status, education, health insurance, and randomization group. Statistical analyses were carried out using SAS 9.4 (Cary, NC).

## Results

Baseline demographic characteristics of the safety-net patients in the sample who completed 90-day follow up surveys are shown in Table 1. Out of 185 participants, 46% were non-Hispanic Black; 23% Hispanic and 28% non-Hispanic White. Most of the participants had some form of insurance, education (High School/GED or below) and were either married/partners or divorced/ separated. Demographic characteristics by receipt of CRC screening show no significant differences between high religious involvement and low religious involvement participants; however, there was a greater percentage of individuals in the low religious involvement group who did not complete CRC screening at 90-day follow up when compared to the high religious involvement group. Among the non-respondents, the results were similar. There were some differences among ethnic groups where 25% of Non-Hispanic Whites and 22% of Hispanics were high religious involvement when compared to 52% of Non-Hispanic Blacks who were categorized as high religious involvement.

Religious characteristics (affiliation, church attendance) were also used to determine participants’ reli gious involvement (R/I) and are listed in Table 2. While 43% of the sample indicated that they attend religious services a few times a month or every week, that percentage dropped to 23% when asked if they participated in other activities in addition to attending services. Participants who responded to the religious affiliation question primarily categorized themselves as either Protestant or Catholic however 30% categorized themselves as “none of the above” while none categorized themselves as Buddhist, Jewish, Atheist or Agnostic.

Adjusting for the effect of the intervention, religious involvement (religious affiliation, church attendance, connection to a higher power) did not have a significant impact (p = 0.35) nor predict the likelihood of individuals to screen for CRC (OR = 1.36) (Table 3). Additional models were built, adjusting further for the potential confounders of age, education, health insurance, marital status (Table 3). There were no significant interactions between the religion/spirituality variables and no potential confounders were found.

## Discussion and Conclusion

Religious involvement (religious affiliation, religious attendance, connection with a higher power) on physical health factors were not significantly related to CRC screening completion nor was it a predictor of CRC screening among this sample of low-income safety-net clinic patients in the Midwest. This finding parallels some of the extant literature that states an inconsistency in the measurement of religious and spiritual factors that pertain to health [32], health outcome and the complexity of multiple societal factors that may predict cancer screening among low-income patients.

The interrelation and overlapping dimensions of religion often create difficulty with measuring this construct and determining the association and/or impact on health behavior outcome measures. Scholars have defined religion as a construct among both individuals and institutions. Religion also is described as an experience that is direct from an “inherited tradition” [32]. Religion also is further defined among sub-populations and is culturally and contextually defined to have an “internal” component or serving as a mechanism that connects an individual to a higher power through communal or worship experiences [33]. Religion in these contexts may have a positive impact on health-related behaviors but also a negative impact. Katz, et al. [34] described this disassociation as “an external locus of control or fatalism (that) takes away the need to seek cancer screening” [34].

The exploratory nature of the study and also measurement of Religious Involvement within a subsample were also limitations of the study. Religious Involvement was not a single measure of religion among this safety net patient population however it did not incorporate other key dimensions that scholars state define this construct *beyond* church attendance and affiliation [21] nor capture functional dimensions of Religious Involvement for a diverse and multi-ethnic sample. Studies show that functional dimensions for Religious Involvement vary for instance between White and Black populations [22]. In addition, only a percentage of individuals from the parent study were captured at 90-day follow up. Although the present sample includes participants who were randomized into the control or experimental arm, a more representative number of individuals may have yielded different results or provided a more precise view of Religious Involvement and its impact of CRC screening among safety-net patients.

The religious landscape of the United States is changing and more individuals are moving away from organized religion; many individuals are leaving their traditional religious backgrounds and are seeking non-denominational institutions or religions [35]. This shifting could also explain the differences between Religious Involvement in our sample and a trend that religious activities and beliefs about connecting to a higher power through religion. Here too, Religious Involvement may be a mediating factor that explains the relationship between how individuals relate to a place of worship and other influential groups or organizations that have impact on cancer screening behavior.

Additional studies should explore the multi-dimensional aspect of Religious Involvement among safety-net clinic patient populations and how the behavior and functional aspects of Religious Involvement impact CRC screening and prevention. These models can be informative in designing interventions and promotion programs to reach and address cancer disparities among low income and minority populations in faith-based settings and those individuals who self-identify as highly religious or religiously affiliated. Results from the analysis may also yield findings that can inform clinicians and public health individuals about the potential impact of religion on cancer screening and how this may affect health outcome among marginalized and uninsured populations.

## Figures and Tables

**Table 1:** Demographic characteristics by CRC screening participation.

	No CRC Screening at 90 Days(N = 98)	CRC Screening at/before 90 days (N = 87)	Total (N = 185)
Gender			
Female	62 (63%)	52 (60%)	114 (62%)
Male	36 (37%)	35 (40%)	71 (38%)
Age			
Mean (SD)	55.92 (5.8)	56.76 (5.7)	56.31 (5.7)
Race/Ethnicity			
Hispanic	19 (19%)	23 (26%)	42 (23%)
Non-Hispanic white	22 (22%)	29 (33%)	51 (28%)
Non-Hispanic black	51 (52%)	35 (40%)	86 (46%)
Non-Hispanic other	6 (6%)	0 (0%)	6 (3%)
Marital Status			
Married/partners	30 (31%)	28 (32%)	58 (31%)
Divorced/separated	37 (38%)	30 (34%)	67 (36%)
Widow/never married/others	31 (32%)	29 (33%)	60 (32%)
Highest Education			
High School/GED/below	56 (57%)	45 (52%)	101 (55%)
College/above	42 (43%)	42 (48%)	84 (45%)
Employment Status			
Full/part-time/seasonal	24 (24%)	23 (26%)	47 (25%)
Looking for work/home/student/retired	54 (55%)	51 (59%)	105 (57%)
Disabled	20 (20%)	13 (15%)	33 (18%)
Insurance Status			
No Insurance	24 (24%)	14 (16%)	38 (21%)
Insurance/Medicare/Medicaid	74 (76%)	73 (84%)	147 (79%)
Randomization Group			
Control	56 (57%)	39 (45%)	95 (51%)
Experimental (Touch 2 Screen)	42 (43%)	48 (55%)	90 (49%)
High Religious/Spiritual (Imputed)			
No	44 (45%)	33 (38%)	77 (42%)
Yes	54 (55%)	54 (62%)	108 (58%)
High Religious/Spiritual (excludes nonresponders)			
No	33 (38%)	21 (28%)	54 (33%)
Yes	54 (62%)	54 (72%)	108 (67%)

**Table 2:** Religious involvement and religious belief question responses by CRC screening participation.

	No CRC Screening at 90 Days (N = 98)	CRC Screening at/before 90 days (N = 87)	Total
How often do you usually attend religious services?			
Never	12 (12%)	16 (18%)	28 (15%)
Occasionally/A few times a year	30 (31%)	18 (21%)	48 (26%)
Once a month	6 (6%)	5 (6%)	11 (6%)
A few times a month	9 (9%)	4 (5%)	13 (7%)
Every week	30 (31%)	36 (41%)	66 (36%)
Unanswered	11 (11%)	8 (9%)	19 (10%)
Besides regular service, how often do you take part in other activities at your place of worship?			
Never	32 (33%)	28 (32%)	60 (32%)
Occasionally/A few times a year	27 (28%)	23 (26%)	50 (27%)
Once a month	7 (7%)	5 (6%)	12 (6%)
A few times a month	6 (6%)	4 (5%)	10 (5%)
Every week	15 (15%)	18 (21%)	33 (18%)
Unanswered	11 (11%)	9 (10%)	20 (11%)
Which of the following best describes the religion you practice?			
Protestant	35 (36%)	22 (25%)	57 (31%)
Catholic	20 (20%)	25 (29%)	45 (24%)
Buddhist	0 (0%)	1 (1%)	1 (1%)
Muslim	2 (2%)	1 (1%)	3 (2%)
Jewish	0 (0%)	0 (0%)	0 (0%)
Atheist	0 (0%)	1 (1%)	1 (1%)
Agnostic	0 (0%)	1 (1%)	1 (1%)
None of the above	29 (30%)	26 (30%)	55 (30%)
Unanswered	12 (12%)	10 (11%)	22 (12%)
Would you describe yourself as a spiritual person?			
Yes	67 (68%)	63 (72%)	130 (70%)
No	14 (14%)	5 (6%)	19 (10%)
Maybe	6 (6%)	10 (11%)	16 (9%)
Unanswered	11 (11%)	9 (10%)	20 (11%)
Religion affects how I make decisions concerning my health.			
Agree	51 (52%)	44 (51%)	95 (51%)
Disagree	35 (36%)	34 (39%)	69 (37%)
Unanswered	12 (12%)	9 (10%)	21 (11%)
I rely on God or a higher power to keep me healthy through prayer.			
Agree	66 (67%)	61 (70%)	127 (69%)
Disagree	21 (21%)	15 (17%)	36 (19%)
Unanswered	11 (11%)	11 (13%)	22 (12%)
I rely on God or a higher power to keep me healthy through prayer and modern medicine?			
Agree	71 (72%)	68 (78%)	139 (75%)
Disagree	16 (16%)	10 (11%)	26 (14%)
Unanswered	11 (11%)	9 (10%)	20 (11%)
I rely on God or a higher power to keep me healthy through alternative medicine.			
Agree	54 (55%)	53 (61%)	107 (58%)
Disagree	30 (31%)	23 (26%)	53 (29%)
Unanswered	14 (14%)	11 (13%)	25 (14%)
I rely on God or a higher power to keep me healthy through prayer and alternative medicine.			
Agree	60 (61%)	56 (64%)	116 (63%)
Disagree	25 (26%)	19 (22%)	44 (24%)
Unanswered	13 (13%)	12 (14%)	25 (14%)
I like receiving cancer information from my place of worship.			
Agree	50 (51%)	42 (48%)	92 (50%)
Disagree	34 (35%)	30 (34%)	64 (35%)
Unanswered	14 (14%)	15 (17%)	29 (16%)
I trust cancer information coming from my place of worship.			
Agree	59 (60%)	46 (53%)	105 (57%)
Disagree	26 (27%)	27 (31%)	53 (29%)
Unanswered	13 (13%)	14 (16%)	27 (15%)
If my religious leader or clergy suggests I get screened for cancer, I am more likely do the screening.			
Agree	57 (58%)	43 (49%)	100 (54%)
Disagree	29 (30%)	31 (36%)	60 (32%)
Unanswered	12 (12%)	13 (15%)	25 (14%)
If I receive cancer information (such as brochures, newsletters) with religious or spiritual messages, this will help me to make a better decision to get screened or to not get screened.			
Agree	54 (55%)	46 (53%)	100 (54%)
Disagree	29 (30%)	28 (32%)	57 (31%)
Unanswered	15 (15%)	13 (15%)	28 (15%)

**Table 3:** Logistic Regression results predicting CRC Screening adjusting for baseline demographic variables, randomization group and high Religious Involvement group (high vs. low) (excludes non-responders).

	Odds Ratio	95% CI Lower Bound	95% CI Upper Bound	P-value
Gender				0.2107
Male	1.00	-	-	
Female	0.65	0.33	1.28	
Age	1.03	0.96	1.10	0.3950
Race/Ethnicity				0.4388
Non-Hispanic white	1.00	-	-	
Hispanic	0.88	0.32	2.38	
Non-Hispanic black	0.51	0.21	1.20	
Non-Hispanic other	< 0.001	-	-	
Marital Status				0.6184
Widow/never married/others	1.00	-	-	
Married/partners	1.24	0.50	3.10	
Divorced/separated	1.52	0.66	3.54	
Highest Education				0.7925
College/above	1.00	-	-	
High School/GED/below	0.91	0.43	1.90	
Employment Status				0.6643
Disabled	1.00	-	-	
Full/part-time/seasonal	1.66	0.55	5.03	
Looking for work/home/student/retired	1.44	0.55	3.75	
Insurance Status				0.8546
No Insurance	1.00	-	-	
Insurance/Medicare/Medicaid	1.10	0.40	3.02	
Randomization Group				0.1489
Control	1.00	-	-	
Experimental (Touch 2 Screen)	1.64	0.84	3.22	
High Religious/Spiritual (not imputed)				0.1021
No	1.00	-	-	
Yes	1.90	0.88	4.08	
